# Miniaturized Tract Percutaneous Nephrolithotomy for Pelvic Stone Management in a Two-Year-Old: A Case Report and Review of Literature

**DOI:** 10.7759/cureus.79475

**Published:** 2025-02-22

**Authors:** Antonio Y Muñoz López, Alejandro Figueroa-Garcia, Said Castro-Zazueta, Carlos Tejeda Andrade, Francisco Gomez Regalado

**Affiliations:** 1 Endourology and Robotic Surgery, Universidad La Salle México, Mexico City, MEX; 2 Urology, Hospital Angeles del Carmen, Guadalajara, MEX

**Keywords:** endourology, kidney stones, miniaturized tract, nephrolithotomy, pediatric

## Abstract

Renal lithiasis in pediatric patients, although rare, affects children of all ages and both genders. Endourology has recently emerged as a safe and effective method for treating stones in children, thanks to advances in equipment and growing experience with retrograde and percutaneous treatments in adults. However, performing percutaneous nephrolithotomy (PCNL) in this age group presents unique challenges due to the smaller anatomical dimensions.

We describe the case of a two-year-old girl with a history of multiple febrile urinary tract infections. A computed tomography (CT) scan shows a stone in the pelvis of the left kidney, measuring 7.43 x 5.64 x 10 mm with a density of 450 HU and no evidence of hydronephrosis. The patient was treated with a single session of miniaturized percutaneous nephrolithotomy (mini-PCNL).

Mini-PCNL presents a safe and effective option for treating kidney stones in pediatric patients. This approach demonstrates a high stone-free rate and low complication incidence, making it a viable alternative for young patients requiring stone removal.

## Introduction

Kidney stones in pediatric patients, although rare, affect children of all ages and both genders [1]. Approximately 50% of kidney stones in children under 10 years of age are attributed to metabolic disorders, inherited metabolic errors, and congenital malformations.

Endourology has recently emerged as a safe and effective method for treating stones in children, thanks to advances in equipment and growing experience with retrograde and percutaneous treatments in adults. Treatment should therefore be individualized, while the patient and their parents should be informed about all available treatment options, which are extracorporeal shock wave lithotripsy (SWL), flexible ureterorenoscopy, retrograde intrarenal surgery (RIRS), and percutaneous nephrolithotomy (PCNL) [2]. However, performing PCNL in this age group presents unique challenges due to the smaller anatomical dimensions.

This case report aims to demonstrate the reproducibility and safety of mini-percutaneous nephrolithotomy (mini-PCNL) using a simplified 0-90° fluoroscopic puncture technique in a two-year-old girl, achieving stone clearance in a single session.

## Case presentation

A two-year-old girl presented to the emergency department multiple times due to febrile urinary tract infections, experiencing four episodes in the last six months. She received empiric antibiotic treatment with cephalosporins, which relieved her symptoms; however, no urine cultures were obtained. She had a history of cardiac surgery for atrial septal defect repair. Physical examination was unremarkable, and the patient weighed 9 kilograms. Urinalysis revealed erythrocyturia, and renal function tests were within normal limits.

**Figure 1:**
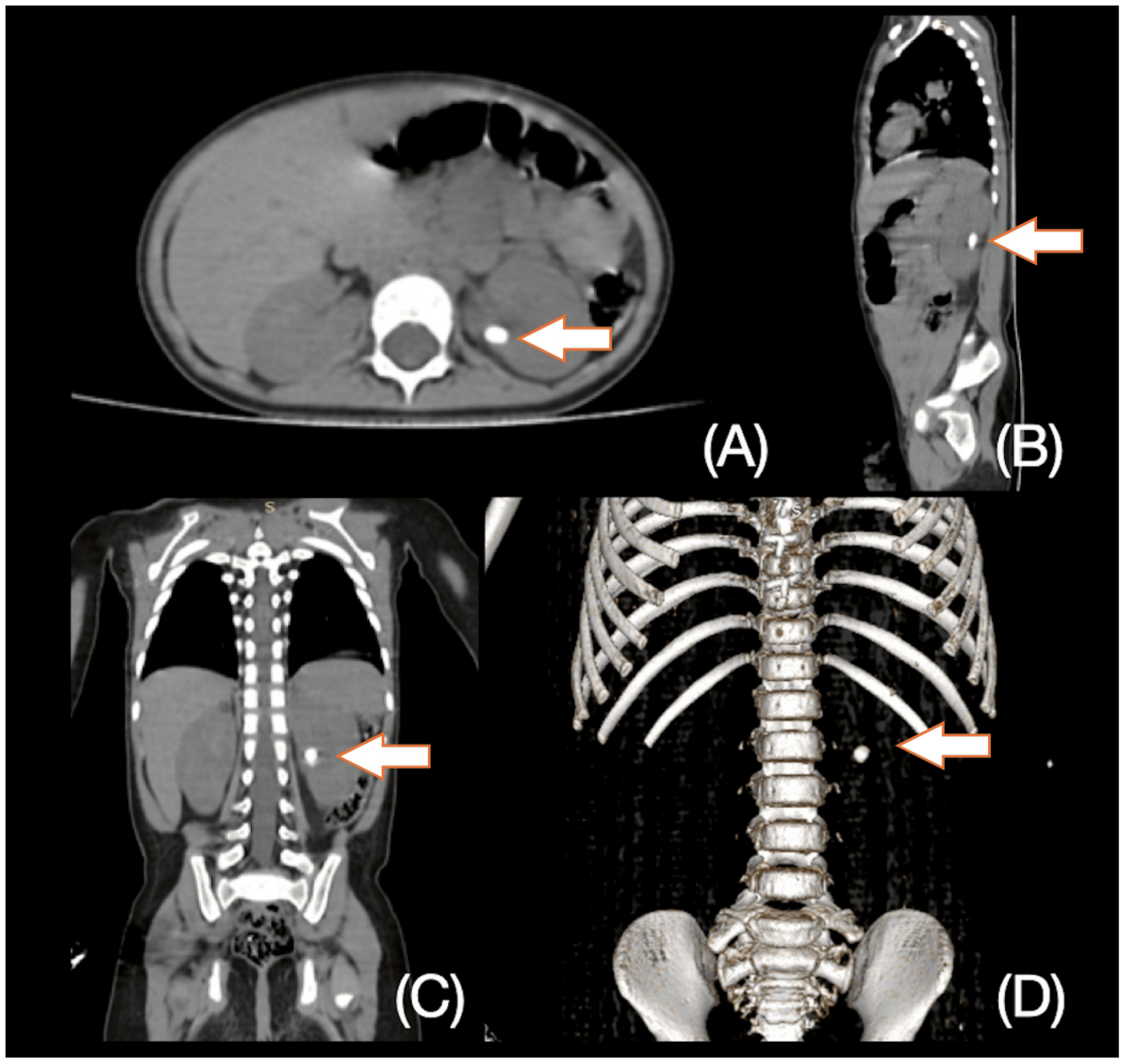
CT scan of the stone disease in the left kidney. A) Axial image, B) sagittal image, C) coronal image, D) 3D reconstruction.

A non-contrast computed tomography (NCCT) scan (Figure 1) identified a calculus in the renal pelvis of the left kidney without any anatomical abnormalities, measuring 7.43 x 5.64 x 10 mm with a density of 450 HU and no evidence of hydronephrosis.

**Figure 2:**
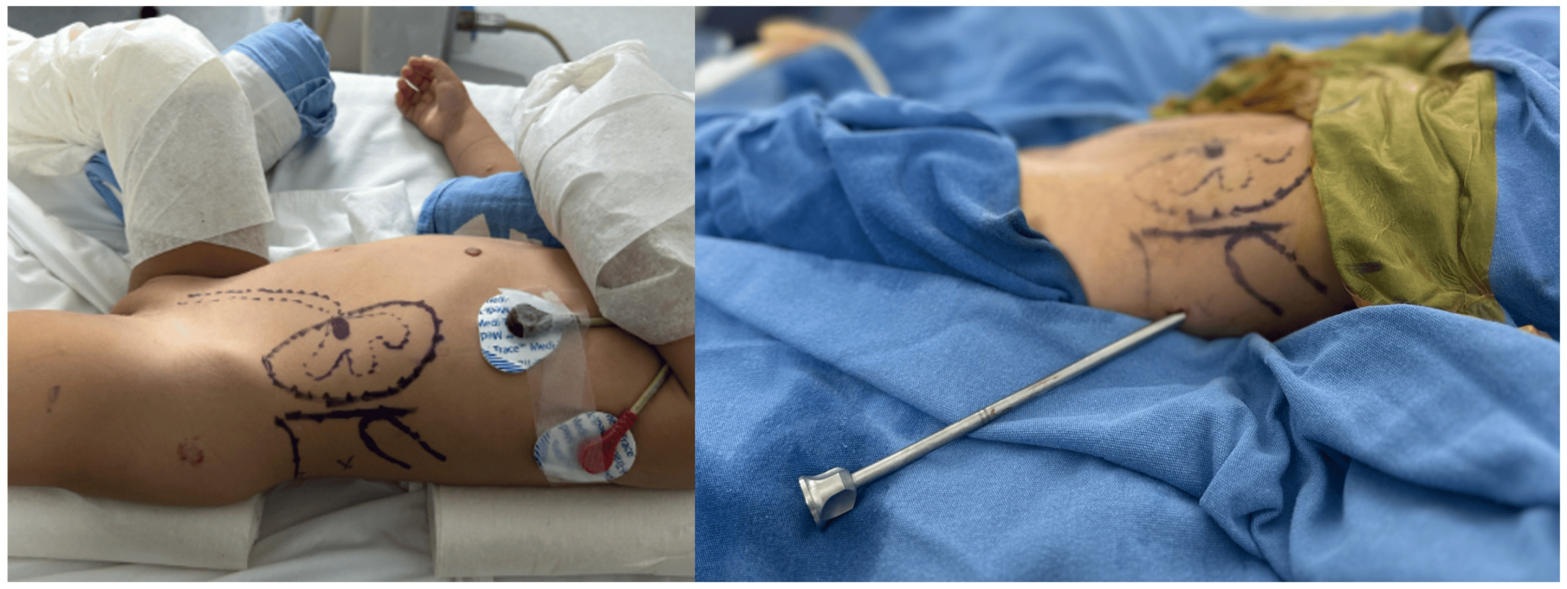
Image showing the patient in the Galdakao-modified position and the sheath of the equipment, also illustrating the marks (posterior axillary line, 12th rib, and iliac crest) to define the safety space for the puncture.

The surgery was performed under general anesthesia with the patient in the supine Galdakao-modified position (Figure 2). A cystoscopy was initially conducted to place an open-end catheter through the left ureter and perform ascending pyelography, assessing the pyelocaliceal anatomy.

**Figure 3:**
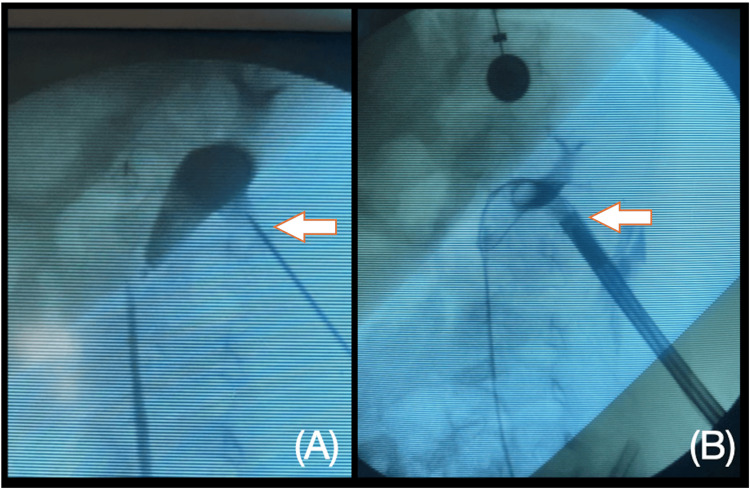
(A) Fluoroscopic projection with ascending pyelography showing the path of the puncture in the inferior calyx. (B) Fluoroscopic projection of the 15 Fr MiniPerc sheath inside the inferior calyx.

**Figure 4:**
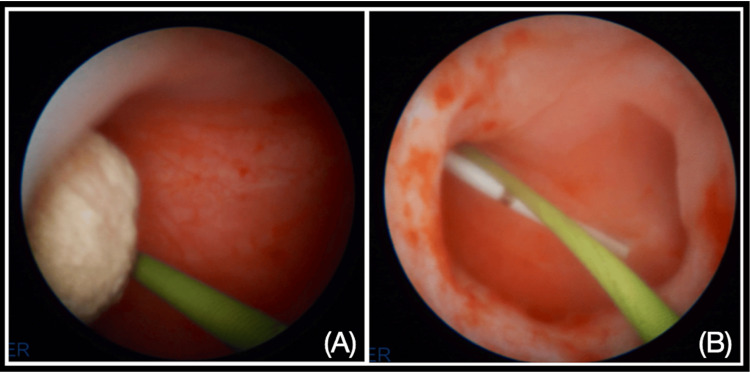
(A) Nephroscopy after performing the tract, showing the stone disease at the renal pelvis and the safety guide. (B) Nephroscopy after performing laser lithotripsy, without stone fragments.

Renal access was achieved using a simplified 0-90 fluoroscopic puncture technique. A single puncture was directed to the lower calyx, followed by the placement of a safety guide and progressive dilation to a 15 Fr MiniPerc sheath (Figure 3). Lithotripsy was performed using the EMS 20W Swiss LaserClast 550µm fiber laser. The total surgical duration was 47 minutes, with 45 seconds of fluoroscopy time. A double-J stent was inserted retrogradely at the procedure's conclusion (Figure 4).

The postoperative period was uneventful, and the patient was discharged 24 hours post-surgery. The stone was sent for analysis, revealing a composition of calcium oxalate and uric acid.

## Discussion

While urolithiasis in children is rare, its global incidence is rising. Over recent years, minimally invasive surgeries have increasingly replaced the previous standard of SWL (extracorporeal SWL) and open surgeries for kidney stones in children, such as PCNL and RIRS [3]. In a systematic review by Qing He et al. in 2019, a total of 13 comparative studies were identified for data analysis. PCNL presented a significantly higher stone-free rate (SFR) compared with SWL. Similarly, the single-session SFR of RIRS was significantly higher than SWL [4].

Mini-PCNL has been demonstrated to be safe and effective for pediatric patients with stone sizes greater than 1 cm, offering high SFRs and a low complication rate, according to a systematic review conducted by Patrick Jones et al. in 2021 [5]. RIRS is also a primary treatment option for pediatric kidney stone disease. Compared to PCNL, RIRS utilizes a natural orifice, making it less invasive and safer, and facilitating postoperative recovery. However, the use of large medical instruments and suboptimal optics technology in RIRS can increase the risk of ureter ischemia, injury, stenosis, and reflux from the bladder and ureter [3].

A meta-analysis by Yuan Yi et al. comparing RIRS to PCNL and miniaturized tracts revealed that while the PCNL group treated significantly larger stones, mini- and micro-PCNL achieved higher SFRs than the RIRS group [3].

In a study by Nevil Akgodan et al., the operative and postoperative outcomes, stone-free status, and complications of SPCNL (standard percutaneous nephrolithotomy) and MPCNL in infants younger than two years were compared. The median age of the 163 patients was 17.3 months (range 7-24 months). Despite the SPCNL group having a lower median stone size, no significant difference was observed between the groups regarding stone size (p = 0.073). The median operative time was significantly longer in the MPCNL group (74.8 minutes) compared to the SPCNL group (62.8 minutes) (p = 0.002). SFRs were 89% for MPCNL and 90.8% for SPCNL, with clinically insignificant residual fragment rates of 11% and 4.6%, respectively (p = 0.064). Fluoroscopy time, nephrostomy withdrawal time, and hospitalization duration were similar between the two groups (p = 0.535, p = 0.253, and p = 0.143, respectively). Postoperative fever rates were also similar (p = 0.504). Although bleeding (6.7% vs. 2.7%) and blood transfusion rates (3.3% vs. 1.4%) were higher in the SPCNL group, these differences were not statistically significant (p = 0.248 and p = 0.420, respectively). Prolonged urinary leakage occurred significantly more in the MPCNL group (8.2%) compared to the SPCNL group (1.1%) (p = 0.026) [6].

In a prospective study by Abuelnaga et al., comparing RIRS and MPCNL outcomes in 60 patients with renal stones between 1 and 2.5 cm, the mean stone size was 1.86 cm for RIRS and 1.69 cm for PCNL (p = 0.449). The PCNL group had significantly longer mean fluoroscopy and hospitalization times. SFRs after a single procedure were 90% for the PCNL group and 83.33% for the RIRS group (p = 0.706). Both groups experienced major complications, while minor complications (Clavien grades 1-3) were 16.66% for PCNL and 13.33% for RIRS. There were no significant differences in operative times between the RIRS and mini-PCNL groups. The mean cost of RIRS was $703.96, compared to $537.03 for mini-PCNL [7].

Hongliang Jia et al. in their retrospective comparative study of super-mini-PCNL against flexible ureteroscopy for the management of upper urinary tract calculus between 1 and 2 cm in children found that the overall SFR was 94.4% for group SMP, and 60.0% for group RIRS in one month after operation (p = 0.001). The re-treatment rate was significantly higher in group RIRS compared to group SMP [20.0% vs 0.0% (p = 0.009)]. The complication rates were 5.6% and 24.0% for groups SMP and RIRS, respectively (p = 0.036) [8], showing in these cases the advantage of the PCNL with a miniaturized tract.

Despite the effectiveness of RIRS in treating renal stones in children, we consider MPCNL a superior approach for stones 1 cm or larger due to its higher likelihood of resolution in a single procedure. A notable disadvantage of RIRS is the increased requirement for JJ stent insertion, either before or after the procedure, necessitating a third procedure for stent removal.

## Conclusions

Mini-PCNL using a simplified 0-90 fluoroscopic puncture technique demonstrates safety and efficacy in treating kidney stones in pediatric patients, even in children as young as two years old. This approach demonstrates a high SFR and low complication incidence, making it a viable alternative for young patients requiring stone removal.

The review of the literature supports the growing preference for mini-PCNL techniques over extracorporeal SWL and RIRS for managing kidney stones larger than 1 cm in children.
